# Timing of Dose Reductions and Survival Outcomes in Metastatic Breast Cancer Patients Treated with Cyclin-Dependent Kinase 4/6 Inhibitors †

**DOI:** 10.3390/curroncol31120548

**Published:** 2024-11-22

**Authors:** Pınar Kubilay Tolunay, Bediz Kurt İnci, Şura Usta, Ali Topkaç, Berkan Karabuğa, Ergin Aydemir, İrem Öner, Büşra Akay Hacan, Öztürk Ateş, Cengiz Karaçin, Ülkü Yalçıntaş Arslan

**Affiliations:** 1 Department of Medical Oncology, Ankara University School of Medicine, Ankara 06590, Turkey; 2 Department of Medical Oncology, Dr. Abdurrahman Yurtaslan Oncology Training and Research Hospital, University of Health Sciences, Ankara 06200, Turkey; bedizkurt@gmail.com (B.K.İ.); sura.oztekin@hotmail.com (Ş.U.); dralitopkac@gmail.com (A.T.); drberkankarabuga@gmail.com (B.K.); drerdemirergin@gmail.com (E.A.); dr_iremoner@hotmail.com (İ.Ö.); busra.akay86@gmail.com (B.A.H.); dr.ozturkates@gmail.com (Ö.A.); cengizkaracin@yahoo.com (C.K.); ulkuarslan63@gmail.com (Ü.Y.A.)

**Keywords:** CDK4/6 inhibitors, breast cancer, dose reduction, survival outcomes

## Abstract

Background/Objectives: Dose reductions in CDK4/6 inhibitors, such as ribociclib and palbociclib, are often necessary due to treatment-related toxicities in patients with advanced breast cancer. This study aims to evaluate the impact of the timing of dose reductions on progression-free survival (PFS) and overall survival (OS) in a real-world cohort. Methods: This single-center, retrospective study included patients treated with ribociclib or palbociclib between 2019 and 2023 at a cancer center in Turkey. Dose reductions due to drug-related toxicities were recorded, and survival outcomes were analyzed. Patients were categorized based on the timing of dose reductions: within the first 3 months (early) and after 3 months (late). Results: Among 392 patients (mean age 57.13 years), 16.8% had dose reductions within 3 months, 21.7% had late dose reductions, and 61.5% had no dose reductions. The mPFS was 14.26 months for early dose reductions, 33.12 months for late dose reductions, and 20.6 months for no dose reductions (*p* < 0.001). The mOS was 37.12 months for early dose reductions, not reached for late dose reductions, and 57.76 months for no dose reductions (*p* < 0.001). Hematological toxicity, primarily neutropenia, was the most common cause of dose reductions. The ECOG performance status, line of therapy, and CDK4/6 inhibitor type were also significant predictors of PFS and OS. Conclusions: Early dose reductions in CDK4/6 inhibitors negatively affect PFS and OS, highlighting the importance of maintaining treatment intensity in the first 3 months. However, late dose reductions do not negatively affect progression-free survival (PFS) or overall survival (OS), with late dose reductions associated with better outcomes. Prospective studies in larger patient populations will further clarify our knowledge on this subject.

## 1. Introduction

Inhibitors of Cyclin-dependent kinases 4 and 6 (CDK4/6), including palbociclib and ribociclib, have revolutionized the treatment of metastatic breast cancer in patients with hormone receptor-positive (HR+) and human epidermal growth factor receptor 2-negative (HER2-) tumors. These drugs work by selectively targeting and inhibiting CDK4 and CDK6, disrupting the cell cycle and subsequently reducing cancer cell proliferation [1]. The PALOMA and MONALEESA trials demonstrated that CDK4/6 inhibitors (palbociclib and ribociclib, respectively), when combined with endocrine therapies like letrozole and fulvestrant, significantly improve progression-free survival in both initial and subsequent lines of treatment. Specifically, in the PALOMA-1, PALOMA-2, and PALOMA-3 studies, palbociclib extended PFS by 10 months, 13.1 months, and 4.9 months, respectively, compared to the control arms (20.2 vs. 10.2 months, 27.6 vs. 14.5 months, and 9.5 vs. 4.6 months, respectively). Additionally, ribociclib, in combination with standard endocrine therapies, has been shown to extend overall survival (OS) by 12.5 months, 12.2 months, and 10.7 months in the MONALEESA-2, MONALEESA-3, and MONALEESA-7 studies, respectively, compared to the control groups (63.9 vs. 51.4 months, 53.7 vs. 41.5 months, and 58.7 vs. 48.0 months, respectively), with hazard ratios of 0.76, 0.73, and 0.76, respectively [2,3,4,5,6,7].

Despite their efficacy, these drugs are associated with side effects, such as neutropenia, which is the most common and significant, affecting over 60% of patients in combination with endocrine therapies. Other prevalent side effects include leukopenia, anemia, fatigue, nausea, diarrhea, increased liver enzymes, QT interval prolongation (mainly with ribociclib), and thrombocytopenia [8,9]. These side effects often lead to dose reductions or even the discontinuation of treatment in clinical practice. Current practice involves a standardized dose reduction strategy in response to adverse events, typically reducing the starting dose in a stepwise manner when significant toxicities occur [10]. The recommended initial dose for palbociclib is 125 mg once daily. In order to manage adverse events of common terminology criteria for adverse events (CTCAE) grade 3 or higher, the dose is first reduced to 100 mg/day and then to 75 mg/day [11]. Similarly, the initial starting dose for ribociclib is 600 mg once daily. In response to significant adverse events, the dose may be decreased in a stepwise fashion to 400 mg/day and subsequently to 200 mg/day [12]. This approach allows for the maintenance of therapeutic benefits while addressing tolerability issues, thereby enhancing patient adherence and overall treatment outcomes.

Previous studies indicate that the survival benefits of these inhibitors are maintained even with modified dosing, underscoring the effectiveness of these agents even at reduced doses [13,14,15]. The impact of the timing of dose reductions on survival outcomes remains unclear, with only a few studies with a limited number of patients evaluating CDK4/6 inhibitor dose reductions as a time-dependent covariate. In one study with 56 patients, those treated with a reduced dose intensity (RDI < 80%) of palbociclib at the 12-week landmark had significantly shorter progression-free survival (PFS) than those with RDI ≥ 80% [16]. Conversely, the PALOMA-2 trial found no significant effect of dose reduction on PFS at 3, 6, and 9 months [9]. Additionally, a recent study from Denmark found a detrimental effect on OS associated with dose reductions within the first 12 weeks of treatment [17]. To the best of our knowledge, there are no studies that compare the overall survival results of dose reductions performed three months before or after with those who have never undergone dose reductions.

This study aims to conduct a more thorough examination of the timing of dose reduction on survival outcomes in patients with HR+ HER2- metastatic breast cancer treated with CDK4/6 inhibitors in combination with endocrine therapy.

## 2. Materials and Methods

This single-center retrospective study examined patients with metastatic breast cancer who were treated with CDK4/6 inhibitors alongside endocrine therapy at Dr. Abdurrahman Yurtaslan Ankara Oncology Training and Research Hospital between January 2019 and December 2023. Data for 424 patients were retrieved from electronic health records. To control for selection bias and ensure a homogenous sample, we excluded 8 patients who started CDK4/6 treatment at a reduced dose, as their initial dosing could affect treatment response comparability. Additionally, we excluded 24 patients with treatment durations of less than 3 months. Including patients with shorter treatment durations could introduce survivorship bias, as early discontinuation may reflect severe adverse events or rapid disease progression. The remaining 392 patients were divided into two groups: those who had dose reductions and those who did not. Among the patients who needed dose reductions, they were further categorized based on whether the dose reductions occurred within the first three months or after three months of initiating therapy.

Due to the reimbursement conditions of general health insurance in Turkey, patients were able to use either ribociclib or palbociclib. Treatments started with the recommended initial dose of palbociclib 125 mg or ribociclib 600 mg for 21 days of each 28-day cycle along with endocrine therapy, and dose modifications were made according to the product information provided by the Turkish Medicines and Medical Devices Agency, in alignment with the recommendations from the European Medicines Agency [11,12].

Data on the demographic and clinical characteristics, treatment line, CDK4/6 inhibitor type and endocrine treatment, timing of dose reductions, drug-related side effects, disease progression, and death time were collected. PFS was defined as the interval from the initiation of CDK 4/6 inhibitors to the date of progression or death, whereas OS was defined as the interval from the initiation of CDK 4/6 inhibitors to the date of death from any cause. Treatment responses were evaluated using the Response Evaluation Criteria in Solid Tumors (RECIST) version 1.1 [18]. Adverse events (AEs) were assessed using the CTCAE version 5.

Statistical Analysis: Demographic and clinical characteristics were summarized using descriptive statistics. The Wilcoxon test was employed to assess repeated nonparametric measurements. To compare numerical continuous variables between two or three groups, Student’s *t*-test and ANOVA were used. The Chi-square test was used to examine relationships between two categorical groups. Survival analyses were conducted using the Kaplan–Meier method, with Cox regression employed for univariate and multivariate analyses of categorical and ordinal variables. To account for potential type I errors due to multiple comparisons, a manual Bonferroni correction was applied. The standard significance threshold (α = 0.05) was divided by the number of comparisons made. Statistical analyses were performed using SPSS version 27.0, with a significance threshold set at *p* < 0.05.

Ethical Considerations: The study received ethical approval from the Clinical Research Ethics Committee of Dr. Abdurrahman Yurtaslan Ankara Oncology Hospital (approval number 2023-11/101, dated 16 November 2023).

## 3. Results

A total of 392 patients with HR+/HER2- metastatic breast cancer who were treated with CDK4/6 inhibitors and endocrine therapy were evaluated. Of the 392 patients, 241 (61.5%) had no dose reductions, 66 (16.8%) experienced dose reductions within the first 3 months, and 85 (21.7%) had dose reductions after 3 months of treatment initiation.

A total of 263 (67.1%) patients were treated with first-line CDK4/6 inhibitors in the metastatic setting. The mean age of the patients was 57.13 years (SD ± 12.38). Patients who underwent dose reductions, either within the first three months or later, were older, with mean ages of 60.0 and 60.35 years, respectively, compared to 55.19 years for those without any dose reductions. This age difference was statistically significant (*p* < 0.001). The ECOG performance status (ECOG PS), presence of comorbidities, menopausal status, recurrent or de novo metastatic status, metastasis sites (visceral vs. bone and/or lymph node), treatment line, CDK4/6 inhibitor type (palbociclib or ribociclib), and endocrine treatment backbone (aromatase inhibitor or fulvestrant) were not significantly different between the groups. Patient characteristics are shown in Table 1. The most prevalent comorbidities included hypertension, diabetes, heart disease, and thyroid diseases.

**Figure 1:**
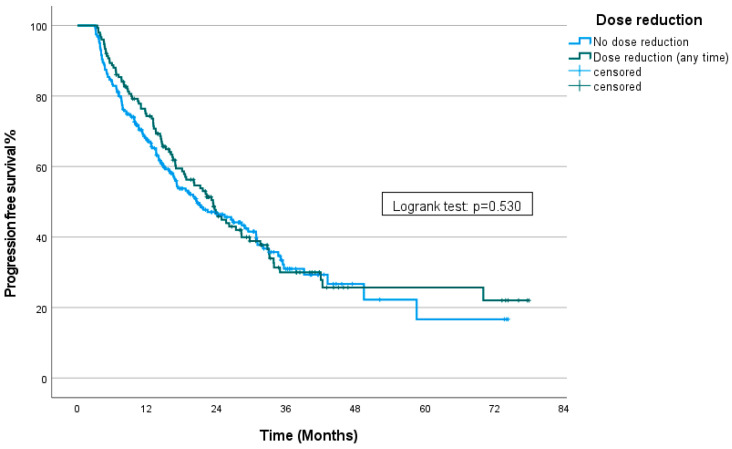
Kaplan–Meier curve showing PFS stratified by the presence of dose reduction.

The median follow-up time was 31.4 months (95% CI: 29.04–33.77). For the entire group, the median progression-free survival (mPFS) was 21.7 months (95% CI: 18.03–25.4). Patients who underwent dose reductions at any time had an mPFS of 23.4 months (95% CI: 19.54–27.25), while those treated with the full dose (no dose reduction) had an mPFS of 20.6 months (95% CI: 14.45–26.7). There was no statistically significant difference between the groups (*p* = 0.53) (Figure 1).

**Figure 2:**
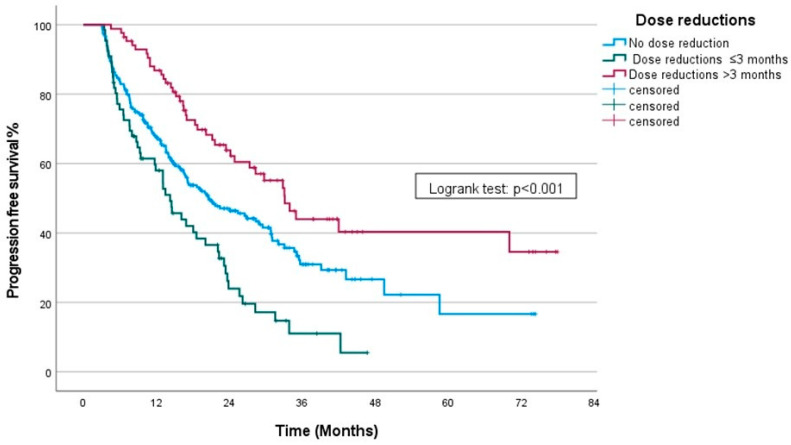
Kaplan–Meier curve showing PFS stratified by timing of dose reductions.

When patients were further grouped by the timing of the dose reduction, those who underwent dose reductions within the first three months had an mPFS of 14.26 months (95% CI: 10.51–18); those who had dose reductions after three months had an mPFS was 33.12 months (95% CI: 27.25–39); and patients without any dose reductions had an mPFS of 20.6 months (95% CI: 14.45–26.74). A statistically significant difference was observed (*p* < 0.001) (Figure 2). Patients who experienced dose reductions after three months of therapy had markedly longer progression-free survival (PFS) compared to those who had dose reductions within the first three months of treatment (*p* < 0.001) and those who did not undergo dose reductions (*p* = 0.002).

The age, ECOG performance status, comorbidity, stage at diagnosis, metastatic status, site of metastasis, line of therapy, choice of CDK4/6 inhibitor, and accompanying endocrine therapy were evaluated using univariate analysis (Cox regression). The ECOG performance status, line of therapy, CDK4/6 inhibitor, and endocrine treatment type were found to significantly impact PFS. When these factors are analyzed in multivariate analysis along with the timing of the dose reduction, the significance of the choice of CDK4/6 inhibitor and hormonal therapy on PFS was lost. However, the ECOG performance status, line of therapy, and timing of the dose reduction continued to have a significant effect on PFS (Table 2).

**Figure 3:**
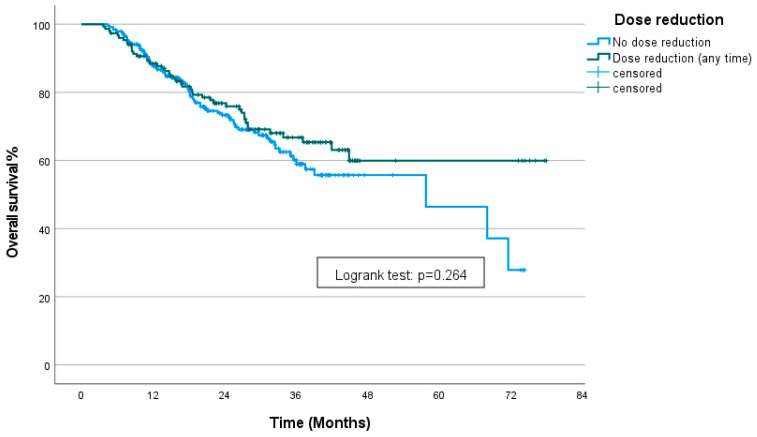
Kaplan–Meier curve showing OS stratified by the presence of dose reduction.

In the entire cohort, the median overall survival (mOS) was 68 months (95% CI: 42.1–94). For patients who underwent dose reductions at any time, the mOS was not reached. Those treated with the full dose (no dose reduction) had an mOS of 57.76 months (95% CI: 29.36–86.15). There was no statistically significant difference between the groups (*p* = 0.26) (Figure 3).

**Figure 4:**
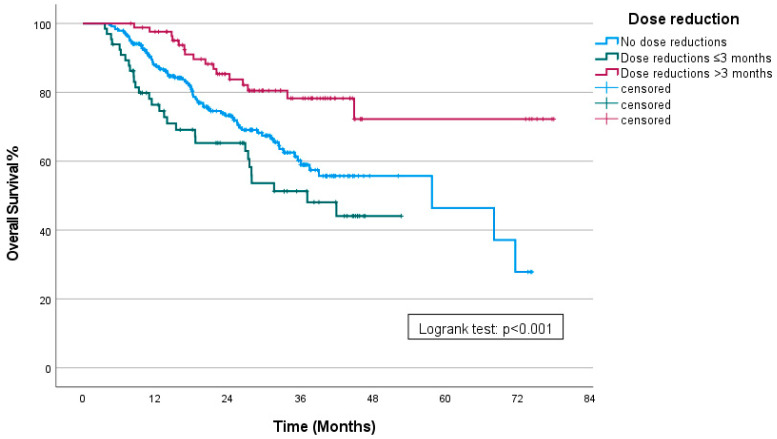
Kaplan–Meier curve showing OS stratified by timing of dose reductions.

When patients were further grouped by the timing of the dose reduction, those who underwent dose reductions within the first three months had an mOS of 37.12 months (95% CI: 20.23–54). For patients who had a dose reduction after 3 months, the mOS was not reached. For patients who did not undergo any dose reduction, the mOS was 57.76 months (95% CI: 29.36–86.15) (*p* < 0.001) (Figure 4). Patients who experienced dose reductions after the first three months of therapy had significantly longer progression-free survival (PFS) compared to those who had dose reductions within the initial three months of treatment (*p* < 0.001) and to patients who did not undergo any dose reductions (*p* = 0.002).

The age, ECOG performance status, comorbidity, stage at diagnosis, site of metastasis, line of therapy, choice of CDK4/6 inhibitor, and combined endocrine therapy were evaluated using univariate analysis (Cox regression). The ECOG performance status, line of therapy, and choice of CDK4/6 inhibitor were found to be significant for overall survival (OS). When these variables were included in a multivariate analysis alongside the timing of dose reductions, the impact of the type of CDK4/6 inhibitor on overall survival (OS) was no longer significant. However, the ECOG performance status, line of therapy, and timing of the dose reduction continued to have a significant effect on OS (Table 3).

Hematologic toxicity, primarily neutropenia, was the most common cause of dose reductions, affecting 114 patients (29.1%). Hepatotoxicity was observed in nine patients (2.3%), followed by cardiologic toxicity in ten patients (2.6%), fatigue in seven patients (1.8%), nephrotoxicity in four patients (1%), diarrhea in two patients (0.5%), and dermatitis in two patients (0.5%). Lung toxicity was reported in one patient (0.3%). Additionally, two patients had dose reductions due to other causes.

## 4. Discussion

Our study aims to assess the effect of the timing of dose reductions in CDK4/6 inhibitors on the survival outcomes of patients with HR+/HER2-metastatic breast cancer. When patients were evaluated based on whether or not they had dose reductions, the difference between the groups was not statistically significant; however, progression-free survival (PFS) was slightly longer in those who underwent dose reduction (23.4 months vs. 20.6 months). Previous analyses of the PALOMA-2, PALOMA-3, and MONALEESA-2, -3, and -7 trials have demonstrated that progression-free survival (PFS) was similar between patients who underwent dose reductions and those who did not, indicating that efficacy is not compromised with dose reductions [19,20,21,22]. PFS for patients with HR+/HER2- mBC treated with CDK4/6is in combination with aromatase inhibitors (AIs) has been reported to range from 20.2 to 27.9 months in clinical trials and from 15.1 to 36.7 months in real-world studies [23,24,25,26,27,28,29,30]. For regimens combining CDK4/6 inhibitors with fulvestrant, PFS ranged from 9.5 to 20.5 months in clinical trials and from 11.6 to 15.7 months in real-world settings [25,26,30,31]. In our study, OS was not reached in patients who had dose reductions, while it was 57.76 months for those receiving the full dose. Although overall survival results are more limited, previous studies have reported ranges between 34.9 months and 63.9 months, with some studies not reaching a final result [2,4,5,6,7]. The line of therapy and the CDK4/6 inhibitor used in treatment influence survival outcomes. Given that approximately one-third of the patients in our study received second-line or later treatment and were treated with palbociclib, our survival results are consistent with previous studies. Briefly, in our study, when patients were grouped by those with dose reductions and those receiving the full dose, progression-free survival and overall survival were not negatively affected.

When we further grouped the patients by the timing of dose reductions, those who had dose reductions after 3 months had significantly better PFS and OS (33.12 and not reached, respectively) compared to those who had no dose reductions (20.6 and 57.76 months, respectively) and those who had dose reductions within the first 3 months (14.26 and 37.12 months, respectively). In our results, patients with dose reductions in the first 3 months had worse survival outcomes. Consistent with our findings, the results showing longer PFS with late dose reductions have also been previously demonstrated with palbociclib and ribociclib [32,33]. It has also been shown that progression-free survival is worse in patients receiving palbociclib who undergo dose reduction within the first 3 months [34]. In a previous study with ribociclib, when comparing those who had dose reductions within the first 3 months to those who did not, it was found that patients who never had a dose reduction had the worst progression-free survival outcomes [32]. In another study from Denmark, which included patients receiving CDK4/6 inhibitors in the first-line setting, it was shown that those who underwent dose reduction within the first 3 months had a shorter treatment duration and overall survival [35].

In our study, along with the timing of the dose reduction, the ECOG performance status, line of therapy, and type of CDK inhibitor were also found to significantly impact both PFS and OS outcomes. As expected, patients with a poorer ECOG performance status had worse survival outcomes, but they made up only around 6% of the total group. Similarly, survival outcomes were worse in patients receiving second-line or later treatments. Approximately 38% of the group was treated with palbociclib, and survival outcomes in this group were poorer. This may be attributed to differences in drug efficacy, usage in later lines of treatment, or the fact that, in clinical practice, patients treated with palbociclib tended to be older and had more comorbidities. When these parameters were included in a multivariate analysis along with the timing of the dose reduction, the timing of the dose reduction continued to have a significant effect on both PFS and OS. These findings highlight that early dose reductions, necessitated by adverse events, can adversely affect survival results. The poorer treatment outcomes in patients with early dose reductions may be attributed to several factors. Early dose reduction may lead to decreased treatment intensity, reducing the treatment’s effectiveness in controlling tumor growth. It can also increase the risk of cancer cells developing resistance to the therapy. Additionally, the need for dose reduction might disrupt treatment continuity and adherence, further diminishing the therapeutic efficacy. After adjusting for patients with late dose reductions, better outcomes are seen in those who had late dose reductions. Thereafter, patients may benefit from lower-dose continued therapy.

The dose reduction rate in our study was 38.5%. In the MONALEESA-2, -3, and -7 trials, 41.8% of dose reductions were attributed to adverse events (AEs) [15]. Similarly, the dose reduction rate in the PALOMA-3 trial was 36% [7]. As expected, the most common toxicity in our study was hematological, primarily neutropenia, which aligns with findings from previous studies.

A limitation of our study is the retrospective design, which may introduce inherent biases. The exclusion of abemaciclib from our analysis, attributable to its non-reimbursement status in our country, represents a notable limitation. Other limitations of our study include the absence of data on treatment interruptions and dose intensity, second dose reductions and treatment discontinuations. Despite its limitations, our study provides single-center, real-world data from a substantial patient cohort.

## 5. Conclusions

CDK4/6 inhibitors have become standard in the first-line treatment for both hormone-positive and HER2-negative metastatic breast cancer. Although the side effects associated with these treatments are well described, real-life data regarding the timing of dose reductions are limited. Our results show that dose reductions, particularly those made after the first 3 months, do not negatively affect progression-free survival (PFS) or overall survival (OS), with late dose reductions associated with better outcomes. However, patients who underwent dose reductions within the first 3 months had worse survival outcomes, likely due to the decreased treatment intensity or severe side effects. Prospective studies in larger patient populations will further clarify our knowledge on this subject.

## Figures and Tables

**Table 1:** Patient characteristics.

Variable	Total	DR < 3 Months	DR > 3 Months	Full Dose	*p* Value
n (n%)	392 (100%)	66 (16.8%)	85 (21.7%)	241 (61.5%)	
Age (mean ± std)	57.13 ± 12.38	60 ± 13.53	60.35 ± 13.17	55.19 ± 11.4	**<0.001**
ECOG PS (n%)					
0–1	369 (94.1%)	61 (92.4%)	79 (92.9%)	229 (95%)	
>1	23 (5.9%)	5 (7.6%)	6 (7.1%)	12 (5%)	0.634
Comorbidity (n%)					
Yes	189 (48.2%)	28 (42.4%)	46 (54.1%)	115 (47.7%)	
No	203 (51.8%)	38 (57.6%)	39 (45.9%)	126 (52.3%)	0.351
Menopausal Status (n%)					
Post	262 (66.8%)	47 (71.2%)	63 (74.1%)	152 (63.1%)	
Pre	130 (33.2%)	19 (28.8%)	22 (25.9%)	89 (36.9%)	0.126
Metastatic Status (n%)					
Recurrent	213 (54.3%)	36 (54.5%)	45 (52.9%)	132 (54.8%)	
De novo	179 (45.7%)	30 (45.5%)	40 (47.1%)	109 (45.2%)	0.958
Metastatic Site (n%)					
Non-visceral	237 (60.5%)	43 (65.2%)	54 (63.5%)	140 (58.1%)	
Visceral	155 (39.5%)	23 (34.8%)	31 (36.5%)	101 (41.9%)	0.47
Treatment Line (n%)					
1st Line	263 (67.1%)	41 (62.1%)	58 (68.2%)	164 (68%)	
≥2nd Line	129 (32.9%)	25 (37.9%)	27 (31.8%)	77 (32%)	0.641
CDK4/6 inhibitor (n%)					
Ribociclib	244 (62.2%)	37 (56.1%)	53 (62.4%)	154 (63.9%)	
Palbociclib	148 (37.8%)	29(43.9%)	32 (37.6%)	87 (36.1%)	0.508
Endocrine backbone (n%)					
Aromatase inhibitor	269 (68.6%)	42 (63.6%)	57 (67.1%)	170 (70.5%)	
Fulvestrant	123 (31.4%)	24 (36.4%)	28 (32.9%)	71 (29.5%)	0.53

DR: Dose reductions.

**Table 2:** Univariate and multivariate analyses to estimate PFS.

Variable	Univariate Analyses			Multivariate Analysis		
	HR	CI 95%	*p* Value	HR	CI 95%	*p* Value
Age	0.99	0.98–1.00	0.49			
ECOG PS						
0–1	2.43	1.51–3.9	**<0.001**	0.39	0.24–0.64	**<0.001**
>1						
Comorbidity						
Yes	1.11	0.86–1.45	0.42			
No						
Menopausal Status						
Post	1.18	0.9–1.56	0.22			
Pre						
Metastatic Status						
Recurrent	0.86	0.66–1.12	0.26			
De novo						
Metastatic Site						
Non-visceral	1.24	0.95–1.61	0.11			
Visceral						
Treatment Line						
1st Line	2.58	1.98–3.37	**<0.001**	2.65	2.02–3.46	**<0.001**
≥2nd Line						
CDK4/6 inhibitor						
Ribociclib	1.46	1.12–1.9	**0.005**			
Palbociclib						
Endocrine backbone						
Aromatase inhibitor	1.55	1.17–2.05	**0.002**			
Fulvestrant						
Dose reduction						
>3 months						
≤3 months	2.91	1.91–4.43	**<0.001**	0.53	0.37–0.76	**<0.001**
Full dose	1.73	1.22–2.47	**0.002**	2.1	1.19–2.28	**0.003**

**Table 3:** Univariate and multivariate analyses to estimate OS.

Variable	Univariate Analyses			Multivariate Analysis		
	HR	CI 95%	*p* Value	HR	CI 95%	*p* Value
Age	1	0.99–1.02	0.5			
ECOG PS						
0–1	0.2	0.12–0.33	**<0.001**	1.19	0.11–0.32	**<0.001**
>1						
Comorbidity						
Yes	1.11	0.77–1.59	0.575			
No						
Menopausal Status						
Post	1.16	0.79–1.68	0.454			
Pre						
Metastatic Status						
Recurrent	0.97	0.68–1.4	0.87			
De novo						
Metastatic Site						
Non-visceral	1.3	0.91–1.87	0.151	0.91	0.77–1.07	0.269
Visceral						
Treatment Line						
1st Line	2.95	2.05–4.25	**<0.001**	0.34	0.24–0.5	**<0.001**
≥2nd Line						
CDK4/6 inhibitor						
Ribociclib	1.58	1.1–2.27	**0.014**			
Palbociclib						
Endocrine backbone						
Aromatase inhibitor	1.35	0.92–1.98	**0.12**			
Fulvestrant						
Dose reduction						
>3 months						
≤3 months	3.38	1.83–6.3	**<0.001**	3.46	1.87–6.41	**<0.001**
Full dose	2.28	1.32–3.92	**0.003**	2.36	1.37–4.06	**0.002**

## Data Availability

Due to patient rights and confidentiality regulations in our country, data sharing cannot be conducted directly, but upon request, data can be sent after consultation with the authors and the ethics committee.
